# Impact of erenumab on acute medication usage and health care resource utilization among migraine patients: a US claims database study

**DOI:** 10.1186/s10194-021-01238-2

**Published:** 2021-04-19

**Authors:** Stewart J. Tepper, Juanzhi Fang, Pamela Vo, Ying Shen, Lujia Zhou, Ahmad Abdrabboh, Mrudula Glassberg, Matias Ferraris

**Affiliations:** 1 Geisel School of Medicine at Dartmouth, Hanover, NH USA; 2 Novartis Pharmaceuticals Corporation, East Hanover, NJ USA; 3 Novartis Pharma AG, CH-4002 Basel, Switzerland; 4 KMK Consulting Inc., Morristown, NJ USA

**Keywords:** Burden, Efficacy, Erenumab, Health care resource utilization, Migraine, Preventive therapies, Real-world evidence

## Abstract

**Background:**

Migraine is one of the leading causes of disability worldwide. Erenumab is a fully human monoclonal antibody that targets the calcitonin gene-related peptide (CGRP) receptor. This study aimed to evaluate real-world evidence on the impact of erenumab on acute medication usage and health care resource utilization (HCRU) among migraine patients.

**Methods:**

This retrospective effectiveness study utilized the US Optum’s de-identified Clinformatics® Data Mart database to identify migraine patients initiating erenumab between May 1, 2018 and September 30, 2019. Patients had to be at least 18 years old, with a minimum of three doses for erenumab in the 6-month post-index period and continuous medical/pharmacy coverage in the 12-month pre- and 6-month post-index period. The date of the first claim for erenumab served as the index date. Use of acute medications overall and at different drug class level, and HCRU were compared during the 6-month pre- vs. post-index period. Impact of erenumab on a *composite endpoint of three possible events*: 1) outpatient visit with a diagnosis of migraine and an associated acute medication claim within 7 days of the visit, 2) hospital admission with a primary diagnosis for migraine, or 3) emergency room visit with a primary diagnosis for migraine (any events that occurred ≤3 days apart were counted only once) was also evaluated.

**Results:**

The analysis included 3171 identified patients. At 6 months, following initiation of erenumab, acute medication use including the number of types of acute medication, number of claims of each medication and % of patients who received acute medication, and HCRU were significantly decreased. For the composite outcome, the mean number of events decreased from 1.03 to 0.77 (rate ratio: 0.75; 95% CI: 0.71 to 0.79; *P* < 0.0001). A decrease in the proportion of patients with any of the three events was also observed (52.7% vs. 39.5%, *P* < 0.0001).

**Conclusion:**

In this retrospective analysis, erenumab was associated with significantly reduced acute medication use and HCRU in a real-world setting, hence significantly reducing the burden of the disease. A composite endpoint could be used as a proxy to evaluate the burden of migraine attacks; however, further research is needed.

**Supplementary Information:**

The online version contains supplementary material available at 10.1186/s10194-021-01238-2.

## Background

Migraine is a common neurological disorder characterized by recurrent attacks of moderate–to–severe headaches and accompanying symptoms (nausea, vomiting, and sensitivity to light, sound, or smell), and is one of the leading causes of disability worldwide [1, 2]. Depending on the frequency and regularity of symptoms, migraine can either be categorized as episodic migraine (EM), defined as < 15 headache days per month, or chronic migraine (CM), defined as ≥15 headache days per month for more than 3 months, of which ≥8 days have features of migraine [2]. In the United States (US), an estimated 19% of individuals in their peak employment years, aged 18–54 years, experience debilitating migraines, such that the condition presents an enormous economic burden for patients, health systems, employers, and society [3]. Pharmacologic treatment of migraine involves both acute and preventive therapy [4–6]. Acute medications aim to provide a sustained pain-free response for a given attack and include migraine-specific medications (triptans and ergots, and more recently lasmiditan and gepants) and nonspecific medications (e.g. nonsteroidal anti-inflammatory drugs [NSAIDs], opioids, and barbiturates) [7]. Although opioids and barbiturates are used clinically, these are not recommended for acute management. Preventive therapies aim to reduce the frequency, severity, duration of attacks, and the impact on quality of life of future migraine headaches. Preventive medications include beta blockers, anticonvulsants, antidepressants, onabotulinumtoxinA (onabotA) injection therapy, and anti-calcitonin gene-related peptide (CGRP) monoclonal antibody (mAb) drugs [8]. Historically, preventive treatment has involved the use of medications, originally developed for other conditions [9], and oral versions have been subject to poor adherence, mainly due to suboptimal efficacy and tolerability [10–12].

Erenumab (in the US, erenumab-aooe) was developed to specifically target the CGRP pathway, which plays a key role in the pathophysiology of migraine [13]. Erenumab, approved in the US for the preventive treatment of migraine in adults in May 2018, is a fully human mAb that selectively binds to and inhibits the CGRP receptor [14, 15]. The efficacy and safety of erenumab in migraine patients have been evaluated in multiple clinical trials [5, 9, 10, 16–20], showing a significant reduction in the mean change from baseline in monthly migraine days [16, 21]. In addition, post-hoc analyses have shown significant improvements in quality of life and disability scales [22]. A recently published real-world audit in ‘hard to treat’ chronic migraine sufferers (all of whom had tried and discontinued multiple migraine preventatives including onabotA), found that erenumab showed significant improvements in the number of headache days, medication use and measures of functional performance [16, 23]. Further real-world evidence on the impact of erenumab on patients with migraine is growing [24, 25]. This study evaluated the real-world impact of erenumab on acute medication usage and health care resource utilization (HCRU) among adult migraine patients in the US. A subgroup analysis reviewed whether erenumab can be effective in patients with more severe migraine phenotypes, including those with a prior unsatisfactory response to onabotA. OnabotA has a relatively higher cost compared to oral migraine preventative medications and has a requirement to be administered in a clinic by trained health care providers (HCPs), making it generally reserved for patients with CM [23].

## Methods

### Study design and data sources

**Fig. 1:**
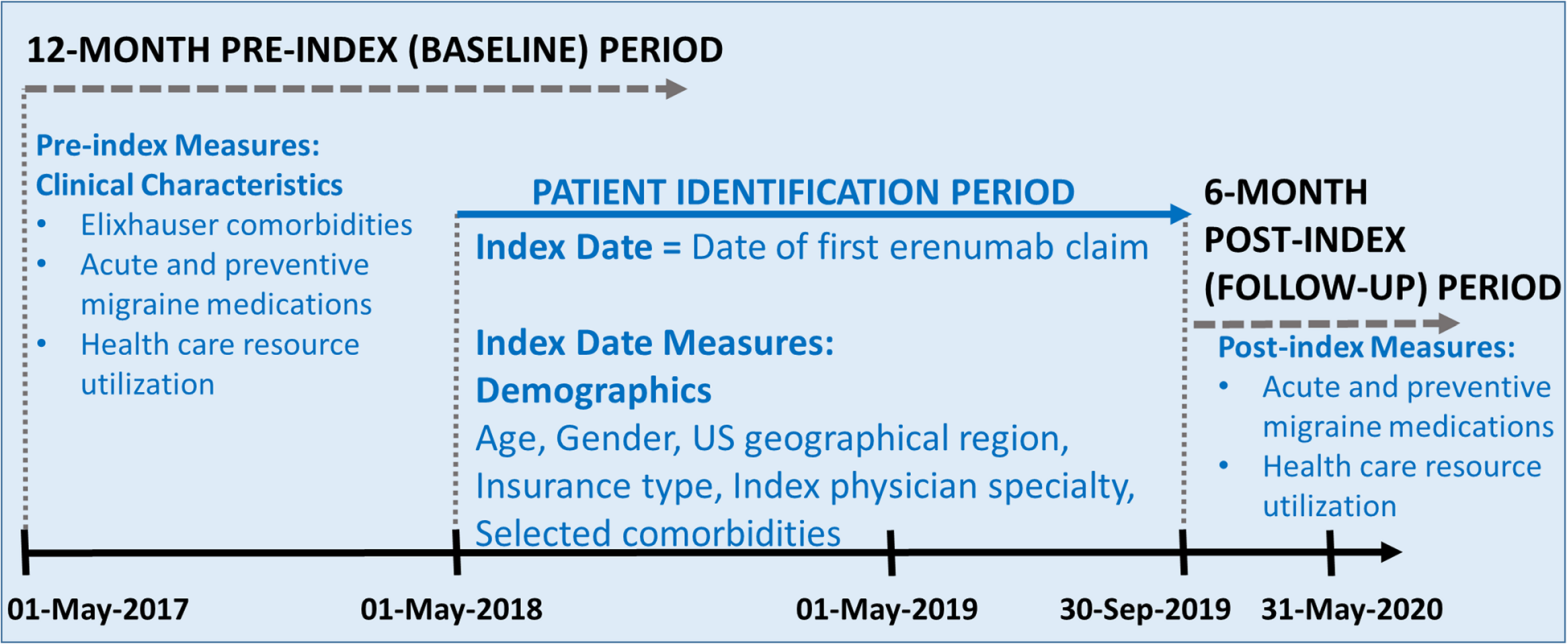
Study Design^a^. ^a^Migraine patients received at least 3 erenumab claims in the 6 months post index period

This study was a retrospective, exploratory treatment effectiveness, non-interventional, observation analysis using Optum’s de-identified Clinformatics® Data Mart (CDM) database, 1st version, 2019 (Fig. 1 and Supp Table 1).

Optum’s CDM is a database of administrative health claims for members of large commercial and Medicare Advantage health plans. The database includes approximately 17–19 million annual covered lives, for a total of over 68 million unique lives over an 11-year period (1/2007 through 9/2019). It is statistically de-identified under the Expert Determination method consistent with the Health Insurance Portability and Accountability Act (HIPAA) and managed according to Optum^®^ customer data use agreements. CDM administrative claims submitted for payment by providers and pharmacies are verified, adjudicated, and de-identified prior to inclusion. These data, including patient-level enrollment information, are derived from claims submitted for all medical and pharmacy health care services. The population is geographically diverse, spanning all 50 US states. As the study used only existing de-identified patient records and analyses of health care claims, data do not meet the definition of human subject research, so Institutional Review Board approval and patient informed consent were not required. No identifiable private information or protected health information was provided. This study was conducted in accordance with the Guidelines for Good Pharmacoepidemiology Practices (GPP) of the International Society for Pharmacoepidemiology (ISPE) 2016, the Strengthening the Reporting of Observational Studies in Epidemiology (STROBE) guidelines, and with the ethical principles laid down in the Declaration of Helsinki.

### Patient selection

Adults (≥18 years of age) with ≥1 prescription fill for erenumab between May 1, 2018 and September 30, 2019 (index window) were identified in the Optum’s CDM database and included for analysis if they met the following study criteria: ≥1 migraine diagnosis between May 1, 2017 and September 30, 2019; index claim (first erenumab claim served as the index date) dispensed from a pharmacy continuously submitting data to Optum’s CDM during the 12 months prior to the index date and 6 months post-index period; and ≥ 3 erenumab doses in the 6 months post-index period. Patients who received other non-erenumab anti-CGRP biologics during the 12-month pre-index or 6-month post-index period were excluded. A planned subgroup analysis further analyzed a cohort of patients receiving prior onabotA therapy during the 12-month pre-index period. The entire study period spanned from May 1, 2017 to March 30, 2020.

### Study measures

#### Patient characteristics

Baseline demographics (age, age group, gender, geographic region, and insurance type) and erenumab prescriber specialty (neurologist/headache specialist, primary care provider [PCP], includes family practice and internal medicine], nurse practitioner [NP]/physician assistant [PA], psychiatrist/psychologist, other specialist, other HCP or unknown/missing) were measured on the index date. Baseline clinical features of 6-month acute, 12-month preventive migraine medication use and comorbidities were measured prior to the index date. Selected comorbidities (such as, anxiety, cardiovascular disease, depression, insomnia, and constipation) were measured during the 12-month pre-index period (not including index date). The Elixhauser comorbidity score was assessed over the12-month pre-index period [26].

#### Treatment patterns of acute and preventive therapies

The number and type of commonly prescribed acute medications (triptans, ergots, NSAIDs, opioids, and barbiturates) in the 6-month pre-index period, and preventive migraine prescription medications (onabotA, tricyclic antidepressants [TCAs], serotonin-norepinephrine reuptake inhibitors [SNRIs], selective serotonin reuptake inhibitors [SSRIs], beta-blockers, calcium-channel blockers, anticonvulsants, and angiotensin converting enzyme [ACE] inhibitors/angiotensin II receptor blockers [ARBs]) in the 12-month pre-index period, were assessed. As some of the acute and preventive medications are also approved for other conditions, a claim associated with a migraine diagnosis was required. The use of non-migraine specific acute medications (NSAIDs, opioids, and barbiturates) required a migraine diagnosis on, or within the 7 days prior to, the medication claim, and the use of non-migraine specific preventive medications required a migraine diagnosis on, or within 14 days prior to, the medication claim with at least 28 days of supply.

### Outcomes measures

The impact of erenumab on acute medication usage during 6 months before and after erenumab treatment among patients with migraine was analyzed as follows: (1) the number of claims on acute medication; (2) proportion of patients with acute medication usage; and (3) the number of different types of acute medications used.

The impact of erenumab on HCRU during 6 months before and after erenumab treatment was measured as follows: (1) All–cause or migraine–specific emergency room (ER) visits or hospitalizations; (2) All–cause or migraine–specific office visits; (3) All–cause or migraine–specific neurologist or headache specialist visits; and (4) All–cause or migraine–specific other outpatient visits.

The impact of erenumab was also measured on a composite endpoint during 6 months before and after erenumab treatment of: (1) outpatient visit with a diagnosis of migraine and an associated acute medication claim; (2) hospital admission with a primary diagnosis for migraine; or (3) ER visit with a primary diagnosis for migraine. Any events that occurred ≤3 days apart were counted only once.

A similar analysis was performed on the onabotA subgroup erenumab cohort.

### Statistical analyses

All analyses were performed using SAS® studio 3.8 (Copyright© 2018, SAS Institute Inc., Cary, NC, USA). Descriptive statistics were used to summarize patient characteristics. To compare 6-months before and after initiation of erenumab data, a negative binomial model with repeated measures was performed for count variables, such as the number of visits and claims, with rate ratios (RR) and 95% confidence interval (CI) calculated; the McNemar test was performed for dichotomous variables; and a proportional odds model with repeated measurement was performed for ordinal variables to assess the odds of having a higher number of different types of acute medications. Means and standard deviations (SD) were reported for continuous measures, and frequencies and percentages were reported for categorical measures.

## Results

### Patient population

**Fig. 2:**
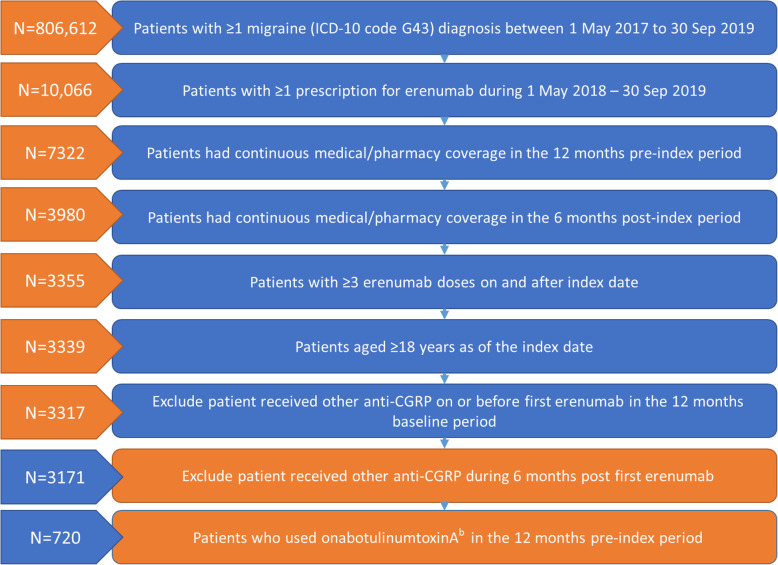
Patient Identification Flowchart^a^. ^a^Associated with a migraine diagnosis ≤14 days post the first migraine diagnosis between 01-May-2018 and 30-Sep-2019.^b^ OnabotulinumtoxinA use requires a migraine diagnosis at most 14 days before claim and days-of-supply ≥28 days. CGRP, calcitonin gene-related peptide

After applying inclusion and exclusion criteria, 3171 patients were identified as being eligible for inclusion in the overall study cohort (Fig. 2). Of these, 720 previous onabotA users were included in the subgroup analysis. Baseline characteristics of the overall and subgroup cohorts are presented in Table 1. Patients in the overall cohort were majority female (84.8%) with a mean [SD] age of 50.7 [13.6] years and 62.5% had CM. Patients were weighted to the Southern (48.4%) and West (21.6%) regions of the US, and almost half (49.5%) had point of service (POS) insurance. The prevalence of the selected comorbidities during the 12-month pre-index period were: anxiety (41.1%), cardiovascular disease (40.9%), depression (40.8%), insomnia (23.1%), and constipation (13.3%). The mean Elixhauser comorbidity score in the 12-month pre-index was 1.70. The score is generated via the summation of points from each disease and the range of possible scores is from − 19 (lesser disease burden) to + 89 (greater disease burden). Elixhauser comorbidities are shown in Supp Table 2.

**Table 1:** Demographics/characteristics of the populations analyzed

	Erenumab cohort (***N*** = 3171)	OnabotA subgroup (***N*** = 720)
Age at index date, mean (SD)	50.7 (13.6)	51.1 (13.2)
Female, n (%)	2689 (84.8)	628 (87.2)
CM in 12 month pre-index period, n (%)	1982 (62.5)	685 (95.1)
**Index physician specialty, n (%)**		
Neurologist/headache specialist	2159 (68.1)	513 (71.3)
General practitioner	439 (13.8)	78 (10.8)
Nurse/physician assistant	293 (9.2)	68 (9.4)
Unknown/missing	154 (4.9)	34 (4.7)
Other specialist	59 (1.9)	11 (1.5)
Other HCP	52 (1.6)	12 (1.7)
Psychiatrist/psychologist	15 (0.5)	4 (0.6)
**Region, n (%)**		
South	1536 (48.4)	301 (41.8)
West	684 (21.6)	205 (28.5)
Midwest	667 (21.0)	146 (20.3)
Northeast	284 (9.0)	68 (9.4)
**Insurance type, n (%)**		
Point of service (POS)	1569 (49.5)	359 (49.9)
Other	741 (23.4)	179 (24.9)
Health maintenance organization (HMO)	518 (16.3)	114 (15.8)
Exclusive provider organization (EPO)	214 (6.7)	38 (5.3)
Preferred provider organization (PPO)	129 (4.1)	30 (4.2)
**Selected comorbidities in 12 month pre-index period (> 10%), n (%)**		
Anxiety	1304 (41.1)	343 (47.6)
CV disease	1298 (40.9)	304 (42.2)
Depression	1295 (40.8)	337 (46.8)
Insomnia	731 (23.1)	176 (24.4)
Obesity	601 (19.0)	141 (19.6)
Fibromyalgia	434 (13.7)	109 (15.1)
Constipation	421 (13.3)	113 (15.7)

*CM* chronic migraine, *CV* cardiovascular, *hcp* health care practitioner, *n* number, *onabotA* onabotulinumtoxinA, *SD* standard deviation, *w/o* without

**Fig. 3:**
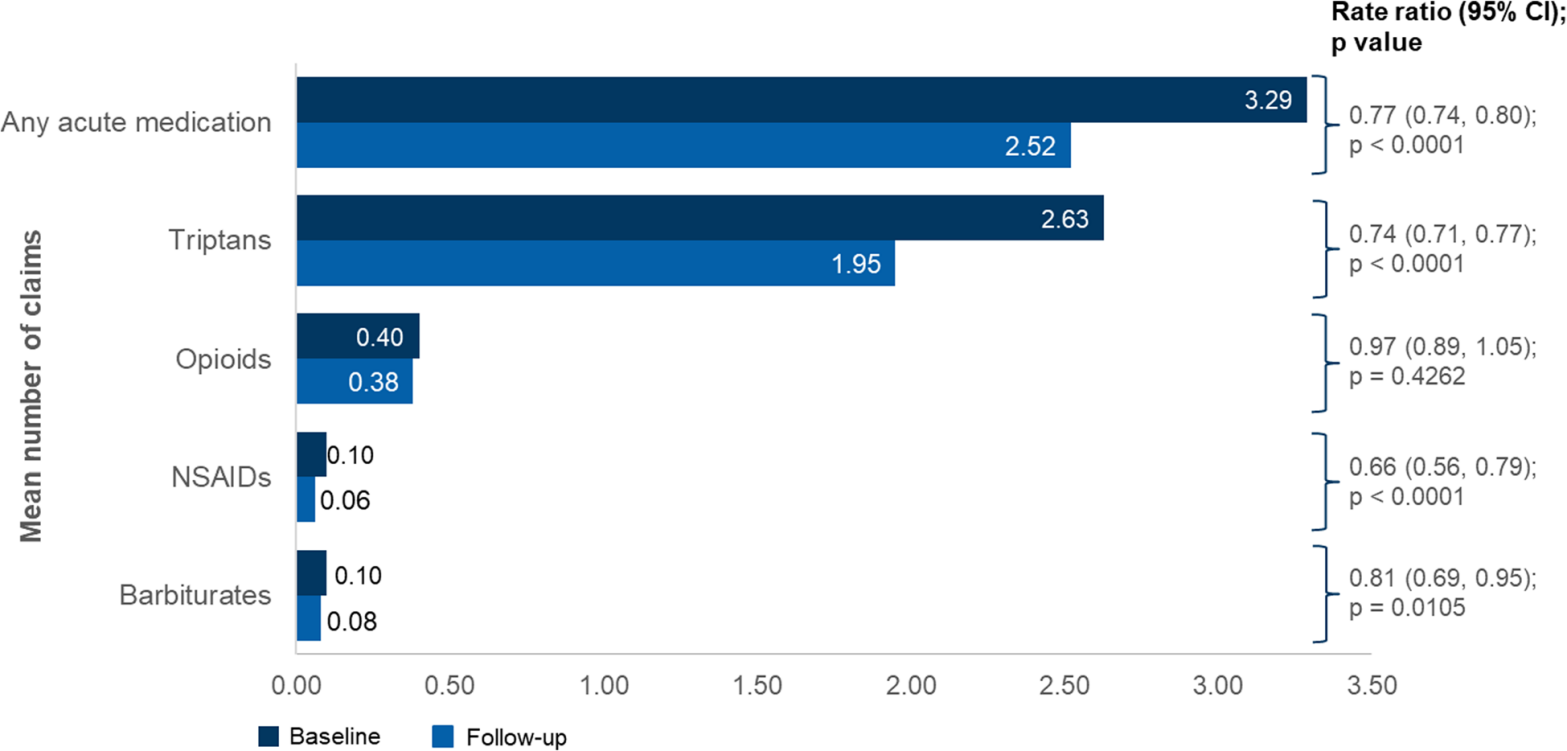
Acute medication use – mean number of claims^a^ in the 183 days before and after erenumab initiation. ^a^Use of non-migraine specific acute medications (NSAIDs, opioids, and barbiturates) required a migraine diagnosis on or before 7 days of the medication claim to proxy migraine-specific acute medication. Negative binomial model with repeated measure was used. CI, confidence interval; ER, emergency room; IPTW, inverse probability of treatment weighting; NSAID, nonsteroidal anti-inflammatory drug; RR, rate ratio

**Fig. 4:**
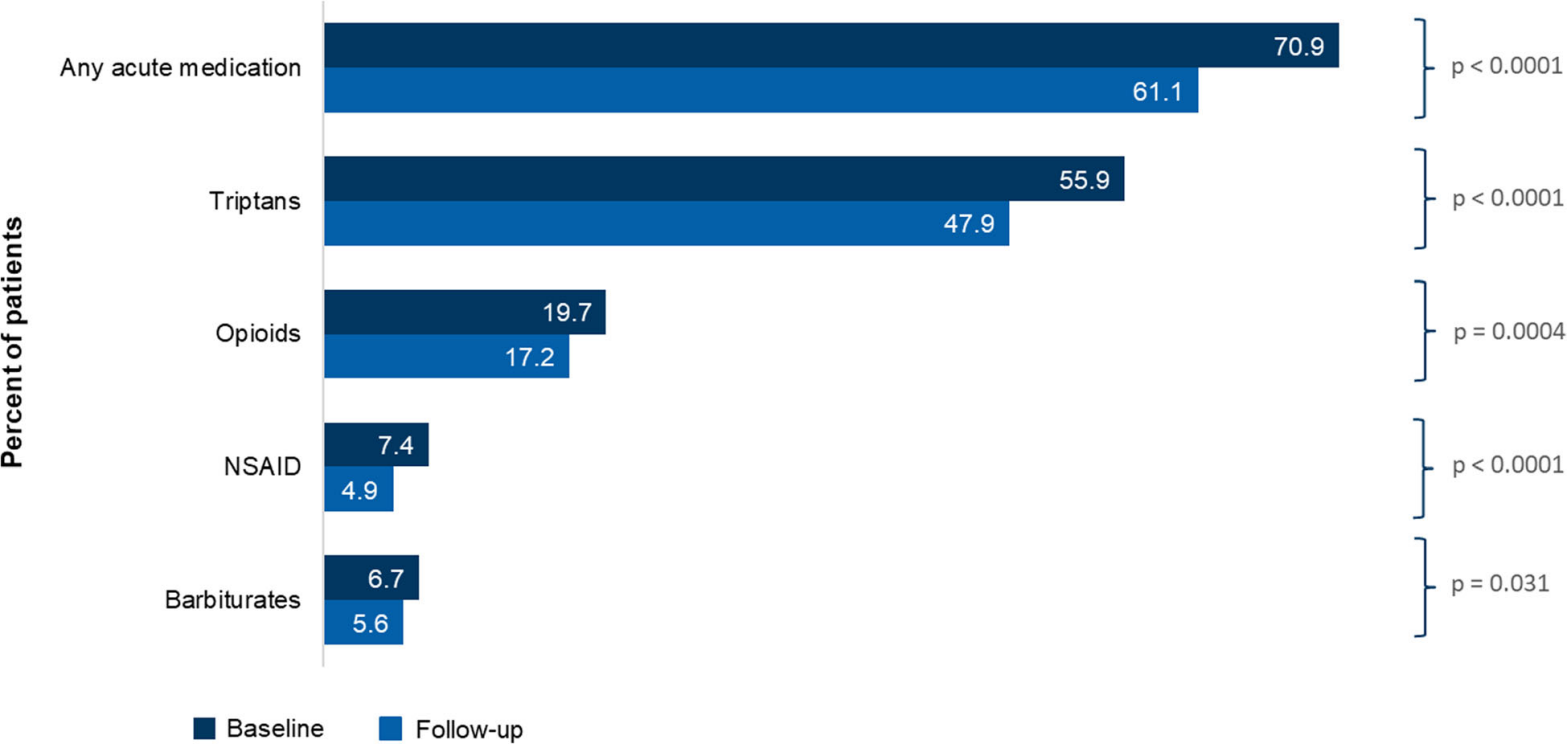
Acute medication use – proportion of patients^a^ in the 183 days before and after erenumab initiation. ^a^Note that the results for ergots are not included due to insufficient data. The McNemar test was performed. NSAID, Non-steroidal anti-inflammatory drug

Neurologists/headache specialists were the most common prescribers of erenumab, initiating 68.1% of the index prescriptions, followed by PCPs (13.8%) and NPs/PAs (9.2%). Prior to initiating erenumab, 70.9% and 71.6% of patients were observed to have had an acute prescription medication for migraine in the 6-month pre-index period or preventive prescription medication in the 12-month pre-index period, respectively. The top four acute medication classes used were triptans (55.9%), opioids (19.7%), NSAIDs (7.4%), and barbiturates (6.7%) (Figs. 3 and 4). The top four preventive medication classes used were anticonvulsants (42.2%), antidepressants (29.0%), onabotA (22.7%), and beta blockers (18.8%) (Supp Table 3). Thirty-five percent of patients were prescribed one preventive migraine drug class, 23.7% were prescribed 2 drug classes, and 12.9% had 3 or more in the 12-months prior to initiating erenumab.

Similar trends were observed within the onabotA subgroup cohort. Patients were a similar age and gender, with 87.2% female and the mean [SD] age 51.1 [13.2] years (Table 1). However, the proportion with a CM without aura diagnosis was significantly higher in this subgroup (62.5% in the overall population and 95.1% in the onabotA cohort). This is expected as onabotA is only approved for CM patients, and therefore, all of these onabotA patients should have had a CM diagnosis. Both groups had similar insurance type (49.9% had POS insurance). The prevalence of selected comorbidities (anxiety, cardiovascular disease, and depression) during the 12-month pre-index period was 47.6%, 42.2%, and 46.8%, respectively. Patients in this cohort experienced higher rates of anxiety (41.1% vs 47.6%) and depression (40.8% vs 46.8%) than in the overall cohort, potentially indicating more debilitating symptoms of attacks.

A slightly higher number of erenumab prescriptions were initiated by a neurologist/headache specialist in the onabotA vs overall cohorts, (71.3% vs 68.1%, respectively), and fewer PCPs initiated erenumab in the onabotA vs overall cohorts (10.8% vs 13.8%, respectively). In 9.4% of cases, erenumab was initiated by NPs/PAs. All patients in this sub cohort (100%) had pre-index use of preventive migraine medications, as they all received onabotA before initiating erenumab. The top four non-onabotA preventive medication classes used were anticonvulsants (48.3%), antidepressants (34.4%), beta blockers (22.8%), and calcium channel blockers (10.1%). Twenty-eight percent of patients were prescribed one preventive migraine drug class, 35.4% were prescribed 2 drug classes, and 36.7% had 3 or more. Overall, 75.4% of patients used at least one acute medication during the 6-month pre-index period. The top acute medications used were triptans (57.2%), opioids (27.8%), and NSAIDs (10.8%) (Supp Fig. 1).

Patients in the onabotA subgroup were prescribed both acute and preventive medications more frequently than the overall population, and were prescribed a higher number of medication classes, indicating that this group experienced migraine episodes of greater severity and potentially greater migraine burden. It is worth noting that the US step edit requirements by payers for approval of erenumab and onabotA generally require failure of two classes of preventive therapy and sometimes, inexplicably, a trial course of triptans. These requirements partially explain the more frequent acute and preventive category prescriptions, but the more frequent prescriptions still suggest greater disease severity and migraine burden or they would not be initiated for the patients.

### Acute medication usage and HCRU

Comparing the 6 months pre-index period and the 6-months post-initiation of erenumab, use of acute medication decreased significantly, with the mean number of claims [SD], declining from 3.29 [4.40] to 2.52 [3.78] (RR 0.77 [95% CI: 0.74–0.80; *p* < 0.0001]), and the proportion of patients using acute medications reduced from 70.9% to 61.1% (*p* < 0.0001) (Figs. 3 and 4). Note the results for ergot use in the 6-month post-index period are not reported due to insufficient data. In addition, after receiving erenumab, patients had significantly lower odds of receiving one or more different types of acute medication vs baseline (OR 0.56; 95% CI: 0.51–0.63; *p* < 0.0001 when counting at the generic drug level and OR 0.57; 95% CI: 0.51–0.64; *p* < 0.0001 when counting at the drug class level (Table 2)).

**Table 2:** Number of acute medications by generic and drug class in the 183 days before and after index

Erenumab cohort	Baseline		Follow-up			
***N*** = 3171	Number	%	Number	%	OR^**a**^ (95% CI)	***P*** value
Number of generic drugs used						
0	872	27.5%	1170	36.9%	0.56 (0.51–0.63)	< 0.0001
1	1333	42.0%	1205	38.0%		
2	610	19.2%	514	16.2%		
3+	356	11.2%	282	8.9%		
Number of drug classes used						
0	872	27.5%	1170	36.9%	0.57 (0.51–0.64)	< 0.0001
1	1545	48.7%	1387	43.7%		
2	560	17.7%	453	14.3%		
3+	194	6.1%	161	5.1%		

*OR* odds ratio, *SD* standard deviation

^a^Proportional odds model was used

**Fig. 5:**
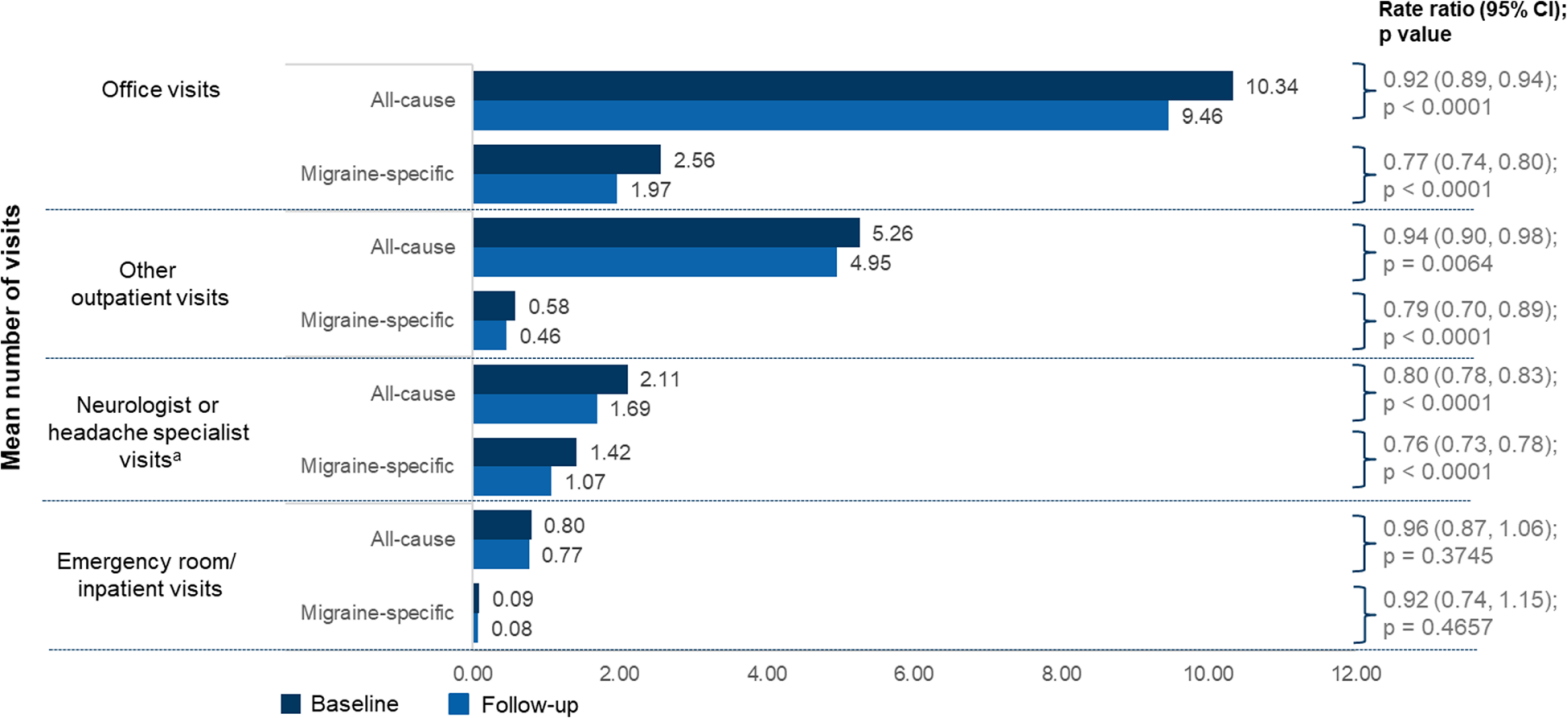
Health care resource utilization – mean number of visits in the 183 days before and after erenumab initiation. ^a^Neurologist or headache specialist visits are a subset of the office visits. Negative binomial model with repeated measure was used. CI, confidence interval; ER, emergency room; IPTW, inverse probability of treatment weighting; NSAID, nonsteroidal anti-inflammatory drug; RR, rate ratio

Similarly, 6 month HCRU decreased significantly, with the mean number of migraine-specific office visits [SD] decreasing from 2.56 [2.68] to 1.97 [2.24] (RR 0.77; 95% CI: 0.74–0.80; *P* < 0.0001), and the proportion of patients with migraine-specific office visits decreasing from 86.2% to 77.6%, *P* < 0.0001 (Fig. 5 and Supp Fig. 2). This significant reduction was similar for neurologist or headache specialist visits (RR 0.76, 95% CI: 0.73–0.78; *P* < 0.0001) and other outpatient visits (RR 0.79, 95% CI: 0.70–0.89; *P* < 0.0001). A small insignificant reduction was also seen in ER/inpatient visits (RR 0.92, 95% CI: 0.74–1.15; *P* = 0.4657).

**Fig. 6:**
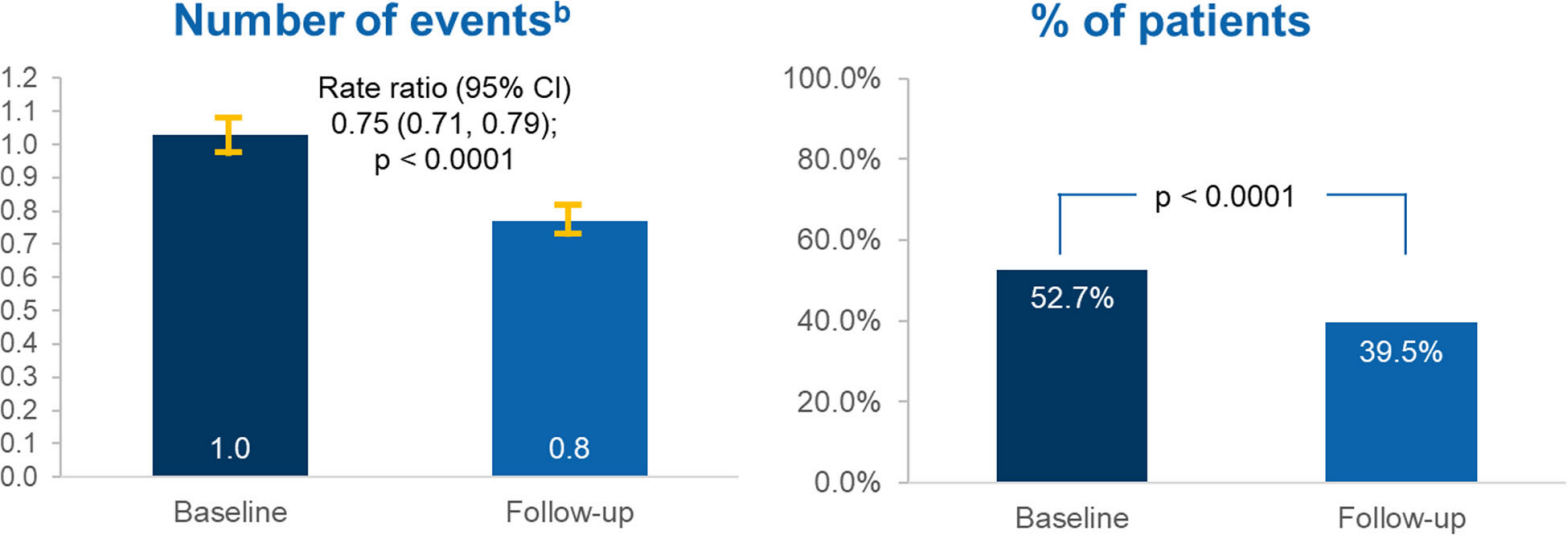
Composite endpoint^a^ in the 183 days before and after erenumab initiation. ^a^Outpatient visit with a diagnosis of migraine and an associated acute medication claim, hospital admission or emergency room visit with a primary diagnosis for migraine. Any events occurred ≤3 days apart were counted only once. McNemar test for binary endpoint and Negative binomial with repeated measure for count data were used. ^b^Number of events are plotted as relative risk and 95% confidence intervals

An exploratory analysis using the composite outcome (comprising [1] outpatient visit with a diagnosis of migraine and an associated acute medication claim, [2] hospital admission with a primary diagnosis for migraine, or [3] ER visit with a primary diagnosis for migraine), found a significant reduction in the mean [SD] number of events, decreasing from 1.03 [1.53] to 0.77 [1.48] (RR 0.75, 95% CI: 0.71 to 0.79, *P* < 0.0001). Moreover, the proportion of patients with any of the three events also significantly decreased from 52.7% to 39.5%, *P* < 0.0001 (Fig. 6).

Analyses of the composite outcome for the onabotA subgroup reflected the results observed in the overall population. In this onabotA subgroup, following the initiation of erenumab, the use of acute medication decreased significantly at 6 months. The mean number of claims [SD] declined from 4.18 [5.57] to 3.31 [5.10] (RR 0.79; 95% CI: 0.74–0.85; *p* < 0.0001), and the proportion of patients using acute medications reduced from 75.4% to 66.5% (*p* < 0.0001) (Supp Fig. 1 and Supp Fig. 3). In addition, patients had significantly lower odds of receiving different types of acute medication vs baseline (OR 0.51; 95% CI: 0.41–0.63; *p* < 0.0001) when counting in generic drug level and OR 0.56; 95% CI: 0.46–0.70; *p* < 0.0001 when counting in drug class level (Supp Table 4)). Following initiation of erenumab, HCRU decreased significantly at 6 months. The mean number of migraine-specific office visits [SD] decreased from 3.99 [3.85] to 2.82 [2.90] (RR 0.70; 95% CI: 0.66–0.75; *P* < 0.0001), and the proportion of patients with migraine-specific office visits decreased from 88.1% to 82.2% (*p* < 0.0001) (Supp Fig. 4 and Supp Fig. 5). A significant reduction in the mean [SD] number of events was observed for the composite outcome from 1.57 [2.08] to 1.17 [2.04] (RR 0.74, 95% CI: 0.68 to 0.82, *P* < 0.0001). Moreover, the proportion of patients with any of the three events also decreased from 62.1% to 48.2%, *P* < 0.0001 (Supp Fig. 6).

## Discussion

We present a look at the real-world effectiveness of a first-in-class anti-CGRP agent (erenumab) approved for the prevention of migraine, on acute medication usage, HCRU, and a composite endpoint reflecting the overall burden of migraine. The population evaluated in these analyses represented patients initiating erenumab early in the post-approval period (with at least one erenumab prescription claim between May 1, 2018 and September 30, 2019) identified from a large US database of administrative health claims for members of commercial and Medicare Advantage health plans. We assessed outcomes in an overall population comprising patients with at least three prescriptions for erenumab therapy. A separate cohort assessed the real-world effectiveness of erenumab in a subgroup of patients receiving prior onabotA therapy during 12-month pre-index period.

Migraine is a complex and long-term disabling neurological disease that is associated with recurrent and often debilitating headaches [27]. A single migraine attack typically disrupts the patient’s life for 4–72 h [28, 29]. Migraines have an impact on patients’ lives well beyond the pain they cause, and the burden of migraine is personal, economic, and societal [26]. Migraine treatment goals are to relieve pain and restore function, reduce the frequency of migraine attacks, prevent transformation of EM to CM, expedite transformation from CM to EM and from acute medication overuse to non-overuse, and manage any existing comorbidities. Treatment decisions should be based on the frequency of migraines (EM or CM), the level of impairment, previous treatment history, and patient preferences [30].

Acute therapies aim to provide a sustained pain-free response from the onset of an attack as quickly as possible (ideally within 2 h from onset), without recurrence, and with minimal adverse events [5, 31–35]. When acute treatments do not effectively control migraine symptoms, patients may escalate care with ER and/or specialist visits. In particular, evidence shows that HCRU and costs are mostly driven by outpatient visits (office and other outpatient visits) [3].

The use of migraine preventive treatments has been shown to reduce undesirable health outcomes and HCRU [36]. The role of preventive medications is to reduce frequency, severity, and duration of attacks, improve responsiveness to acute therapy, reduce migraine-related disability, and favorably impact the quality of life of migraine headaches [9–11]. Studies show that established migraine preventive options prior to 2018 were associated with high discontinuation rates due to tolerability issues and lack of efficacy [13–15]. In the US, a 2015 study reported that up to 83% of patients had discontinued the traditional preventive therapies within 12 months after initiation [11, 37–39]. A large proportion of patients prescribed preventive medications often have a history of prior medication failures, switching treatments, relying solely on acute medications, and overuse of acute medications. This can lead to increases in migraine frequency, transformation of EM to CM, and occurrence of acute medication overuse headache (MOH) [40, 41]. With effective preventive therapy, patients would not need to take as many acute medications, and the efficacy of acute medications would be improved; this would reduce the risk of acute medication tolerance/dependence, and MOH [42].

The results of this report suggested that over a period of 6 months, the initiation of erenumab significantly decreased the use of acute medication (RR 0.77; 95% CI: 0.74–0.80; *p* < 0.0001). Patients were less likely to require different classes of acute medications after being treated with erenumab (OR 0.57; 95% CI: 0.51–0.64; *p* < 0.0001). Similar outcomes were observed in the onabotA subgroup analysis (RR for pre- and post-index use of acute medication: 0.79 (95% CI: 0.74–0.85; *p* < 0.0001, and OR 0.56; 95% CI: 0.46–0.70; *p* < 0.0001 when counting in different classes of acute medications).

Effective preventive therapy, therefore, has the potential to lessen the global economic burden of migraine by reducing outpatient visits and improving participation in everyday activities such as school and work, thereby reducing absenteeism and increasing productivity [43–45]. The economic burden of migraine, with health care and lost productivity costs associated with migraine, is estimated at $36 billion annually in the US [3, 46]. Given the widespread prevalence of migraine headache and the associated high rates of resource utilization, clinicians should make concerted efforts to reduce HCRU [4].

In this study, the initiation of erenumab significantly decreased HCRU of migraine-specific office visits (RR 0.77; 95% CI: 0.74–0.80; *P* < 0.0001) and all-cause office visits (RR 0.92; 95% CI: 0.89–0.94; *p* < 0.0001). Similar outcomes were observed in the onabotA subgroup HCRU analysis (migraine-specific office visits: RR 0.70; 95% CI: 0.66–0.75; *p* < 0.0001 and all-cause office visits: RR 0.89; 95% CI: 0.85–0.93; *p* < 0.0001). This reduction of at least 23% in HCRU is a very meaningful change, given the widespread prevalence of migraine and the associated high rates of resource utilization.

Erenumab significantly reduced the composite endpoint, which indicates an overall reduction in the burden of the disease in patients with migraine. The proportion of patients with any of the three events comprising the composite significantly decreased from 52.7% to 39.5%, *P* < 0.0001, and the mean [SD] number of events decreased from 1.03 [1.53] to 0.77 [1.48]. A composite endpoint such as the one reported in this study may potentially be used as a proxy to evaluate migraine attacks, although further research is needed.

### Limitations

The use of claims data is subject to several limitations, and Optum’s CDM is a US database. The results gained from claims analysis apply only to the insured population in the US, which may not be generalizable to the overall population, or to the international population. Claims data in the US are currently dependent on professional International Classification of Diseases (ICD) coding and not the International Classification of Headache Disorders, 3rd Edition (ICHD-3) and, therefore, some diagnoses may be missed, different professional types may have different coding patterns, and not all coding may be accurate. Further limitations of prescription claims data are that over-the-counter (OTC) exposure is missed, and a prescription does not always guarantee patient administration. Patients receiving erenumab from the free drug program are also not captured in the Optum’s de-identified CDM database; thus, it is impossible to ascertain whether the first erenumab claim is truly indicative of the first time erenumab is used by a given patient. In these cases, the actual effectiveness might be larger than observed because baseline data may have been favorably affected by prior exposure to erenumab.

One further limitation is that this is a single arm study and it is difficult to account for regression to the mean.

In spite of these limitations, claims data are a valuable resource for exploratory analyses of a variety of health services research questions.

## Conclusions

This research provides insight into real-world effectiveness of a first-in-class CGRP pathway-targeted mAb therapy for the prevention of migraine. Erenumab significantly reduces acute medication use on both number of claims and number of different classes, and HCRU in the real-world setting, hence significantly reducing the burden of the disease. A significant reduction of 25% on the composite endpoint of the outpatient visits with an acute medication claim and migraine-specific ER or inpatient visits shows the overall benefit of erenumab in the real-world. The personal, economic, and societal burden of migraine can be eased by improving acute care therapy and earlier treatment commencement of effective preventive therapy. A composite endpoint could be used as a proxy to evaluate migraine attacks, although further research is needed.

## Supplementary Information

**Additional file 1: Supp Fig 1**. Acute medication use – proportion of patients^a^ in the 183 days before and after erenumab initiation. **Supp Fig. 2**. Health care resource utilization – proportion of patients in the 183 days before and after erenumab initiation. **Supp Fig. 3**. Acute medication use – mean number of claims^a^ in the 183 days before and after erenumab initiation. **Supp Fig. 4**. Health care resource utilization – mean number of visits in the 183 days before and after erenumab initiation. **Supp Fig. 5**. Health care resource utilization – proportion of patients in the 183 days before and after erenumab initiation. **Supp Fig. 6**. Composite endpoint^a^ in the 183 days before and after erenumab initiation. **Supp Table 1**. Diagnosis codes. **Supp Table 2**. Elixhauser Comorbidities (≥5%). **Supp Table 3**. Baseline preventive drugs used in the 12 month pre-index period. **Supp Table 4**. Number of acute medications by generic and drug class in the 183 days before and after erenumab initiation

## Data Availability

All data generated or analysed during this study are included in this published article [and its supplementary information files].

## Abbreviations

ACE: Angiotensin converting enzyme
ARB: Angiotensin II receptor blockers
CDM: Clinformatics^®^ Data Mart
CGRP: Calcitonin gene-related peptide
CI: Confidence interval
CM: Chronic migraine
EM: Episodic migraine
EPO: Exclusive provider organization
ER: Emergency room
GPP: Guidelines for Good Pharmacoepidemiology Practices
HCP: Health care professional
HCRU: Health care resource utilization
HIPPA: Health Insurance Portability and Accountability Act
HMO: Health maintenance organization
ICD: International Classification of Diseases
ICHD-3: International Classification of Head- 508 ache Disorders, 3rd Edition
ISPE: International Society for Pharmacoepidemiology
mAb: monoclonal antibody
MOH: Medication overuse headache
NSAID: Nonsteroidal anti-inflammatory drugs
NP: Nurse practitioner
onabotA: onabotulinumtoxinA
OR: Odds ratio
OTC: over-the-counter
PCP: Primary care providers
PA: Physician assistants
POS: Point of service
PPO: Preferred provider organization
RR: Rate ratio
SD: Standard deviation
SNRI: Serotonin-norepinephrine reuptake inhibitors
SSRI: Selective serotonin reuptake inhibitors
STROBE: Strengthening the Reporting of Observational Studies in Epidemiology
TCA: Tricyclic antidepressants
US: United States
