# Supplementary material for: Impact of erenumab on acute medication usage and health care resource utilization among migraine patients: a US claims database study
Source: J Headache Pain. 2021 Apr 19;22(1):27. doi: 10.1186/s10194-021-01238-2 (PMC8054394; doi:10.1186/s10194-021-01238-2)
Supplement: Supplementary file 1 — Additional file 1: Supp Fig 1. Acute medication use – proportion of patientsa in the 183 days before and after erenumab initiation. Supp Fig. 2. Health care resource utilization – proportion of patients in the 183 days before and after erenumab initiation. Supp Fig. 3. Acute medication use – mean number of claimsa in the 183 days before and after erenumab initiation. Supp Fig. 4. Health care resource utilization – mean number of visits in the 183 days before and after erenumab initiation. Supp Fig. 5. Health care resource utilization – proportion of patients in the 183 days before and after erenumab initiation. Supp Fig. 6. Composite endpointa in the 183 days before and after erenumab initiation. Supp Table 1. Diagnosis codes. Supp Table 2. Elixhauser Comorbidities (≥5%). Supp Table 3. Baseline preventive drugs used in the 12 month pre-index period. Supp Table 4. Number of acute medications by generic and drug class in the 183 days before and after erenumab initiation [file 10194_2021_1238_MOESM1_ESM.docx]

### **Supp Fig 1.** Acute medication use – proportion of patients^a^ in the 183 days before and after erenumab initiation

| **OnabotulinumtoxinA Users Subgroup (n=720)** |
| --- |
| **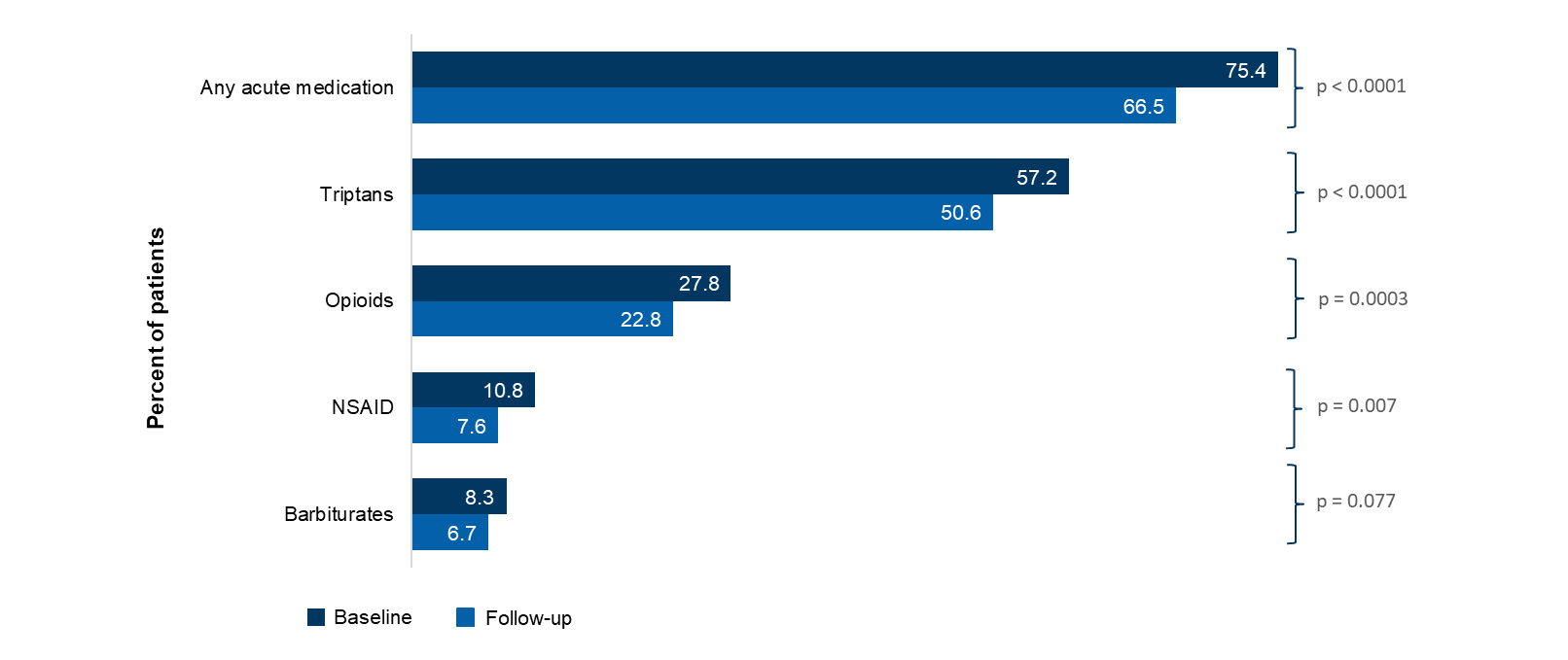** |

^a^Note that the results for ergots are not included due to insufficient data.

The McNemar test was performed.

NSAID, Non-steroidal anti-inflammatory drug.

### **Supp Fig 2.** Healthcare resource utilization – proportion of patients in the 183 days before and after erenumab initiation

| **Overall Population (n=3171)** |
| --- |
| **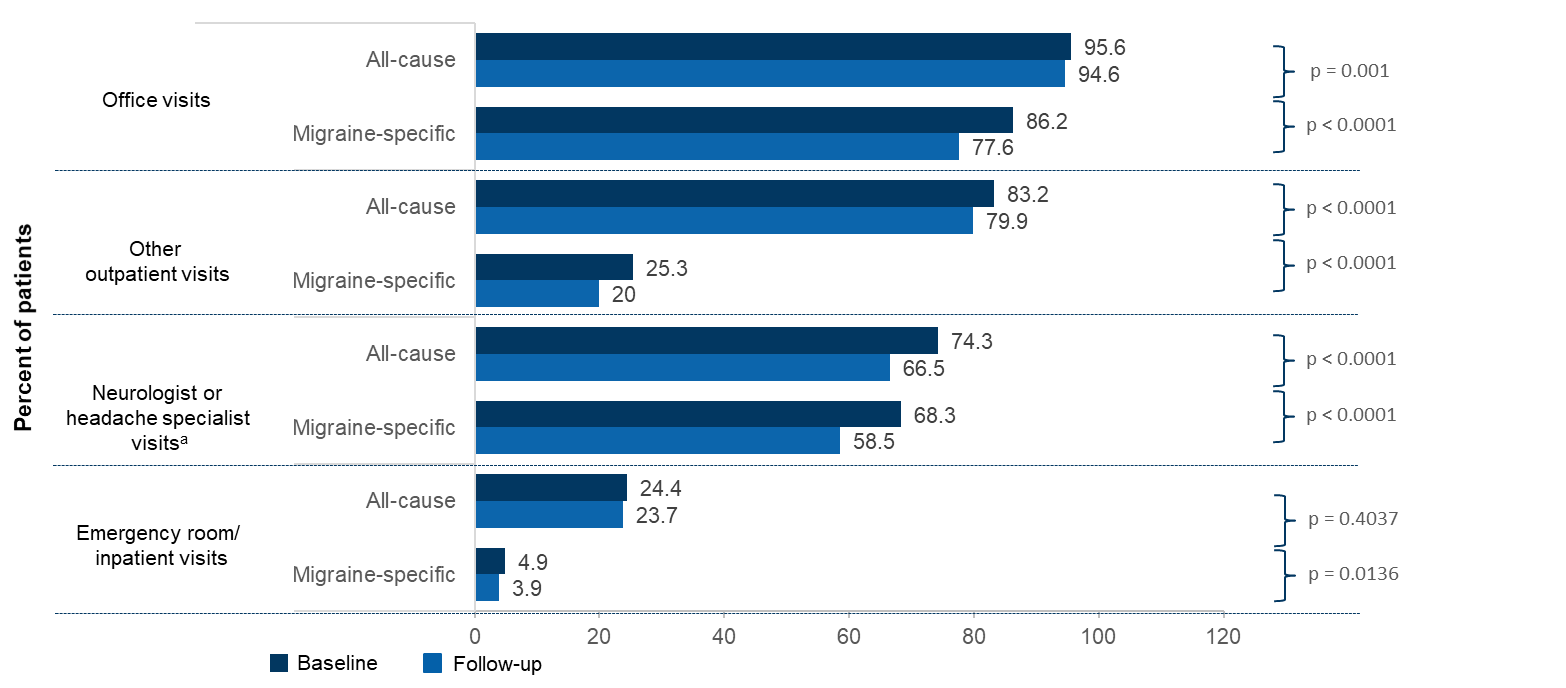** |

^a^Neurologist or headache specialist visits are a subset of the office visits.

The McNemar test was performed.

### **Supp Fig 3.** Acute medication use – mean number of claims^a^ in the 183 days before and after erenumab initiation

| **OnabotulinumtoxinA Users Subgroup (n=720)** |
| --- |
| **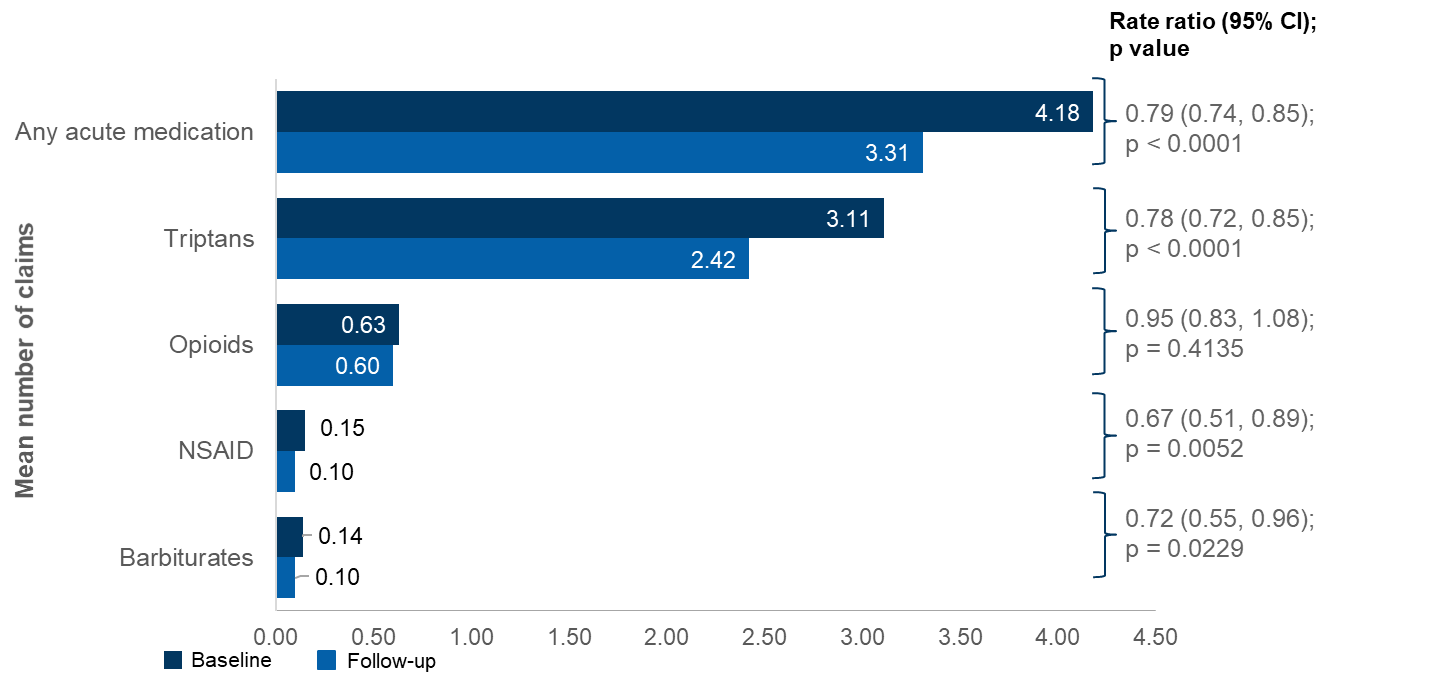** |

^a^Use of non-migraine specific acute medications (NSAIDs, opioids, and barbiturates) required a migraine diagnosis on or before 7 days of the medication claim to proxy migraine-specific acute medication.

Negative binomial model with repeated measure was used.

CI, confidence interval; ER, emergency room; IPTW, inverse probability of treatment weighting; NSAID, nonsteroidal anti-inflammatory drug; RR, rate ratio.

### **Supp Fig 4**. Healthcare resource utilization – mean number of visits in the 183 days before and after erenumab initiation

| **OnabotulinumtoxinA Users Subgroup (n=720)** |
| --- |
| **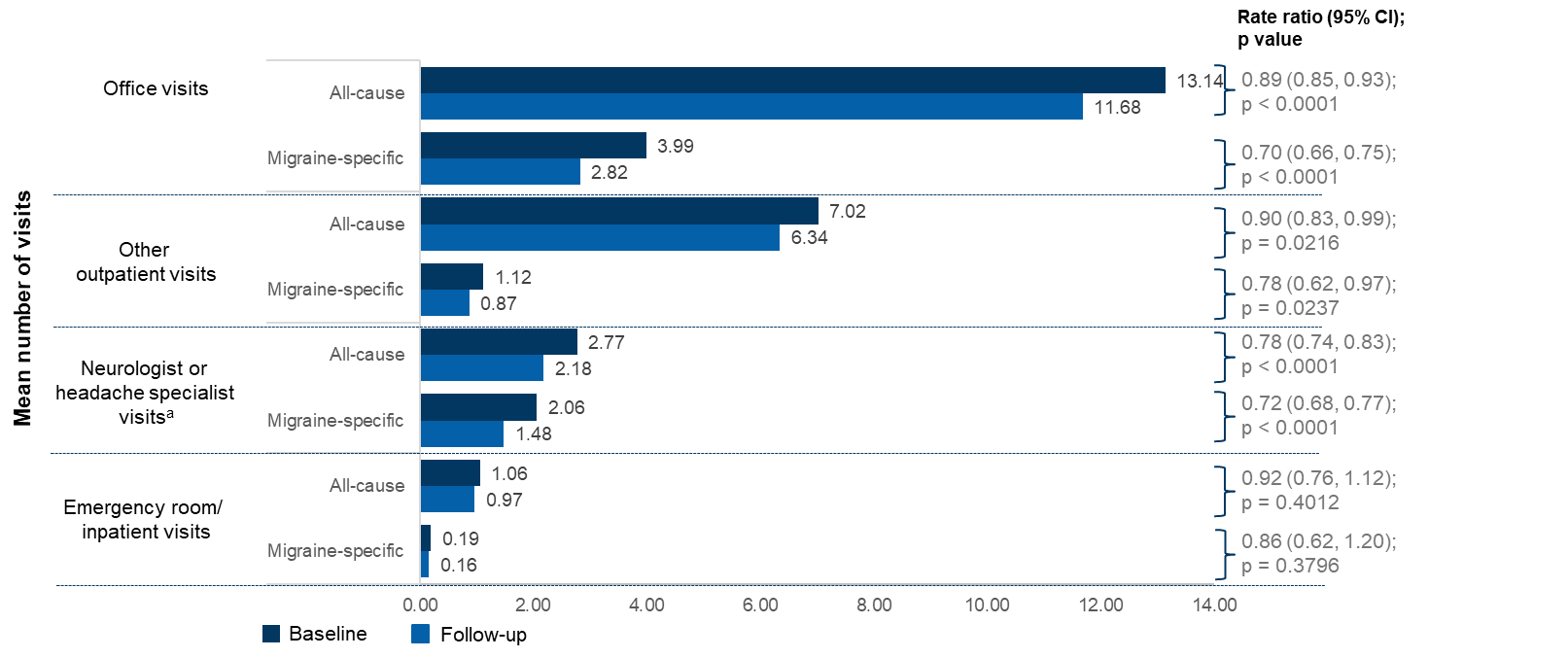** |

^a^Neurologist or headache specialist visits are a subset of the office visits.

Negative binomial model with repeated measure was used.

CI, confidence interval; ER, emergency room; IPTW, inverse probability of treatment weighting; NSAID, nonsteroidal anti-inflammatory drug; RR, rate ratio.

### **Supp Fig 5.** Healthcare resource utilization – proportion of patients in the 183 days before and after erenumab initiation

| **OnabotulinumtoxinA Users Subgroup (n=720)** |
| --- |
| **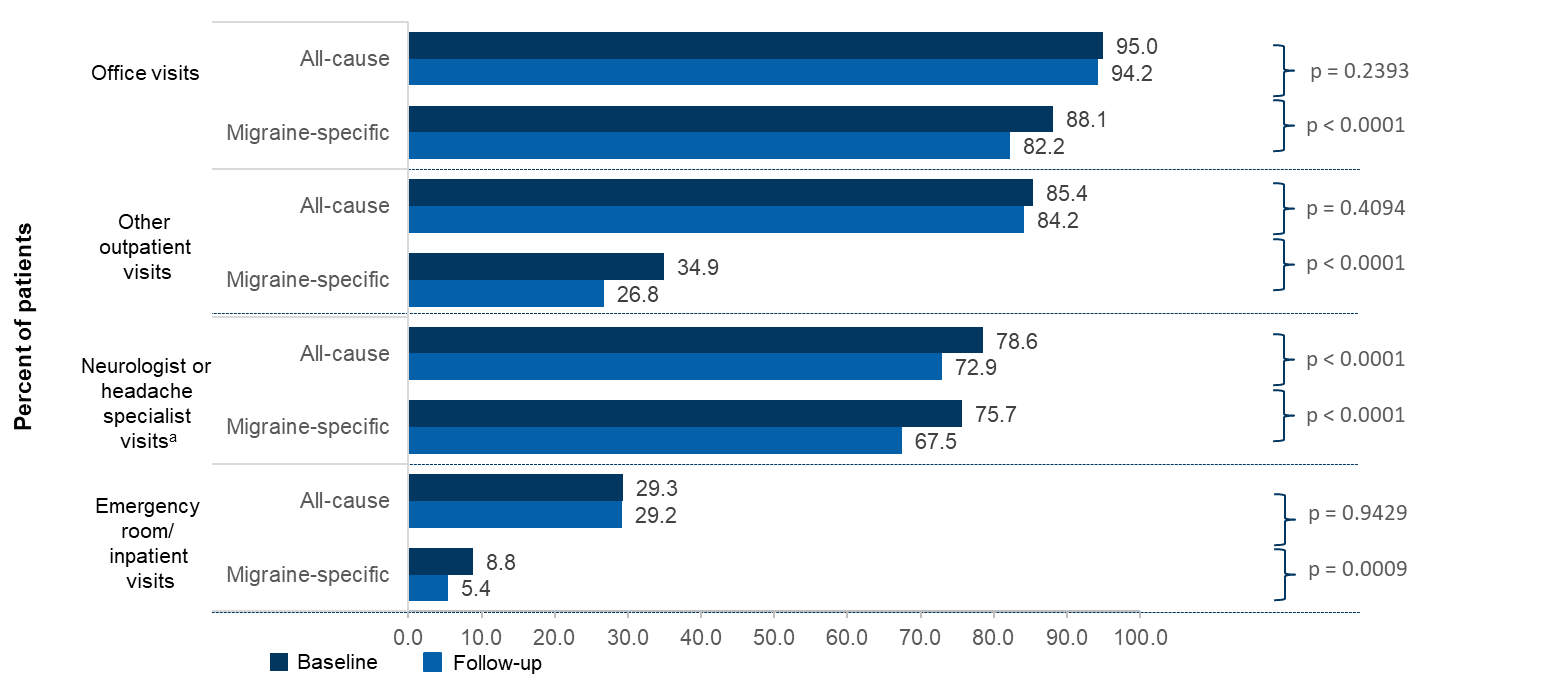** |

^a^Neurologist or headache specialist visits are a subset of the office visits.

The McNemar test was performed.

### **Supp Fig 6**. Composite endpoint^a^ in the 183 days before and after erenumab initiation

| **OnabotulinumtoxinA Users Subgroup (n=720)** |
| --- |
| **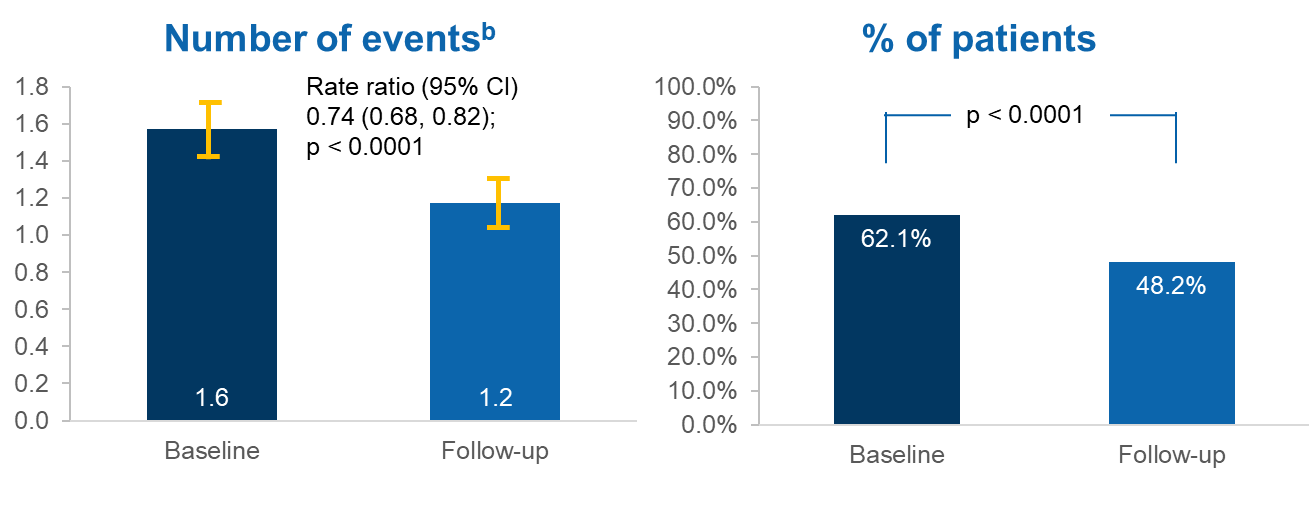** |

^a^Outpatient visit with a diagnosis of migraine and an associated acute medication claim, hospital admission or emergency room visit with a primary diagnosis for migraine. Any events occurred ≤3 days apart were counted only once. McNemar test for binary endpoint and Negative binomial model with repeated measure for count data were used.

^b^Number of events are plotted as relative risk and 95% confidence intervals.

### **Supp Table 1.** Diagnosis codes

| **Disease** | **ICD10** |
| --- | --- |
| Stroke | G450, G451, G452, G453, G458, G459, H340, I60, I61, I62, I63 |
| CV Disease | E85, I0981, I10, I11, I110, I12, I120, I13, I130, I132, I15, I16, I20, I21, I22, I25, I2511, I25110, I25111, I25118, I25119, I252, I257, I2570, I25700, I25701, I25708, I25709, I2571, I25710, I25711, I25718, I25719, I2572, I25720, I25721, I25728, I25729, I2573, I25730, I25731, I25738, I25739, I2575, I25750, I25751, I25758, I25759, I2576, I25760, I25761, I25768, I25769, I2579, I25790, I25791, I25798, I25799, I34, I340, I35, I350, I351, I36, I37, I480, I481, I482, I4891, I50, I700, I739, N183, N184, N185, N186 |
| Depression | F0631, F0632, F251, F313, F314, F315, F3175, F3176, F32, F33, F341, F4321, F4323 |
| Anxiety | F064, F40, F41, F4322, F4323 |
| Insomnia | A8183, F510, G470, Z7381 |
| Epilepsy | G40 |
| Constipation | K560, K564, K566, K567, K590, K5939, 3E1H78Z. |
| Gastroparesis | K3184 |
| IBS | K58, K580, K581, K582, K588, K589 |
| Endometriosis | N80 |
| Obesity | E6601, E6609, E661, E662, E668, E669 |
| Migraine | G43 |
| CM w/o aura | G437 |
| Brain Injury | S0 |
| Menstrual Migraine | G4382, G4383 |
| Fibromyalgia | M797 |

CM, chronic migraine; CV, cardiovascular; IBS, irritable bowel syndrome; w/o, without.

Note: The medication codes are provided in the accompanying excel sheet.

### **Supp Table 2**. Elixhauser Comorbidities (≥5%)

| **Elixhauser Comorbidities with Any Comorbidities: N (%)** | **2588 (81.6%)** |
| --- | --- |
| **Elixhauser disease groups:** | **N (%)** |
| Depression | 1325 (41.8%) |
| Uncomplicated Hypertension | 1143 (36.0%) |
| Hypothyroidism | 775 (24.4%) |
| Chronic Pulmonary Disease | 761 (24.0%) |
| Obesity | 690 (21.8%) |
| Cardiac Arrhythmias | 448 (14.1%) |
| Rheumatoid Arthritis/Collagen Vascular Diseases | 432 (13.6%) |
| Uncomplicated Diabetes | 421 (13.3%) |
| Fluid and Electrolyte Disorders | 350 (11.0%) |
| Complicated Diabetes | 292 (9.2%) |
| Other Neurological Disorders | 283 (8.9%) |
| Deficiency Anemia | 274 (8.6%) |
| Drug Abuse | 266 (8.4%) |
| Peripheral Vascular Disease | 215 (6.8%) |
| Renal Failure | 193 (6.1%) |
| Liver Disease | 191 (6.0%) |
| Valvular Disease | 187 (5.9%) |
| Complicated Hypertension | 181 5.7%) |

The Elixhauser comorbidity index measures the baseline comorbidity based on inpatient and outpatient administrative claims data.

### **Supp Table 3.** Baseline preventive drugs used in the 12 month pre-index period

|  | **Erenumab cohort**  **N = 3171** | **OnabotA subgroup N = 720** |
| --- | --- | --- |
| Number of preventive drug classes used, n (%) |  |  |
| 0 | 902 (28.5) | 0 |
| 1 | 1110 (35.0) | 201 (27.9) |
| 2 | 751 (23.7) | 255 (35.4) |
| 3+ | 408 (12.9) | 264 (36.7) |
| Preventive medications used in 12 months pre-index period, n (%) | | |
| Anticonvulsant | 1338 (42.2) | 348 (48.3) |
| Antidepressant | 920 (29.0) | 248 (34.4) |
| OnabotA | 720 (22.7) | 720 (100) |
| Beta blocker | 597 (18.8) | 164 (22.8) |
| Calcium channel blocker | 274 (8.6) | 73 (10.1) |
| ACE inhibitor | 61 (1.9) | 19 (2.6) |

ACE, Angiotensin-converting enzyme; onabotA, onabotulinumtoxinA.

### **Supp Table 4.** Number of acute medications by generic and drug class in the 183 days before and after erenumab initiation

| **OnabotA subgroup** | **Baseline** | | **Follow-up** | | |  |  |
| --- | --- | --- | --- | --- | --- | --- | --- |
| **N = 720** | **Number** | **%** | **Number** | **%** | | **OR^a^**  **(95% CI)** | **P value** |
| Number of generic drugs used | | | | | | | |
| 0 | 170 | 23.6% | 228 | 31.7% | | 0.51  (0.41-0.63) | <0.0001 |
| 1 | 260 | 36.1% | 268 | 37.2% | |  |  |
| 2 | 152 | 21.1% | 125 | 17.4% | |  |  |
| 3+ | 138 | 19.2% | 99 | 13.8% | |  |  |
| Number of drug classes used | | | | |  |  |  |
| 0 | 170 | 23.6% | 228 | 31.7% | | 0.56  (0.46-0.70) | <0.0001 |
| 1 | 307 | 42.6% | 305 | 42.4% | |  |  |
| 2 | 166 | 23.1% | 123 | 17.1% | |  |  |
| 3+ | 77 | 10.7% | 64 | 8.9% | |  |  |

OR, odds ratio; SD, standard deviation.

### ^a^Proportional odds model with repeated measure was used.
